# A case series on minor incision cataract surgery in small pupil without any aids

**DOI:** 10.22336/rjo.2024.65

**Published:** 2024

**Authors:** Mary Stephen, Nirupama Kasturi, Jayasri Periyandavan, Arun Sahi

**Affiliations:** Department of Ophthalmology, JIPMER, Puducherry, India

**Keywords:** small pupil, pupil expanders, small incision cataract surgery, phacoemulsification, blindness

## Abstract

Cataract is the leading cause of treatable blindness worldwide, and cataract surgery complications leading to blindness are a common cause of preventable blindness. All surgeons aim to obtain a good pupil dilation intra-operatively to ease the surgery. The small pupil is often challenging and contributes to intra and post-operative complications. Phacoemulsification, though, has many options to tackle small pupil. The same options cannot be employed in small incision cataract surgery, especially using mechanical pupil expanders. Small incision cataract surgery is very commonly performed in developing countries. The authors describe a case series of small pupil cataracts managed successfully without the use of any secondary aids to cause pupil dilatation and explain the techniques employed to manage small pupil while performing Small incision cataract surgery. With correct techniques, operative complications of small pupil can be minimized, and small incision cataract surgery is still a helpful option, especially in resource-limited settings, to provide an excellent visual recovery.

## Introduction

Cataract surgery-related complications leading to vision loss are common worldwide, and the rate is being controlled with recent advances. However, poor pupil dilation and intra-operative miosis have a higher risk of posterior capsular rent and vitreous loss, often leading to further complications and poor visual recovery [1]. Small pupil can be due to various reasons, including local and systemic causes. A few common reasons include pseudoexfoliation, uveitis, trauma, glaucoma, previous ocular surgery, and systemic causes include diabetes mellitus, and intake of agents like Tamsulosin (alpha 1a receptor antagonist) leading to intra-operative floppy iris syndrome characterized by progressive pupil constriction and iris bellowing and complications in those patients can be up to 12% [2]. Various pre- and intra-operative modalities can help achieve good pupil dilation, ranging from NSAIDs days before surgery to intracameral adrenaline/phenylephrine, stretch pupilloplasty, sphincterotomy, and pupil expansion devices. Most pupil expansion devices are safer in phacoemulsification than in small incision cataract surgery, and other mechanical methods can be associated with increased postoperative inflammation, photophobia, and irregular pupil. The authors report six cases of small pupil cataract managed well by performing small incision cataract surgery without mechanical aids.

## Case 1

**Fig. 1:**
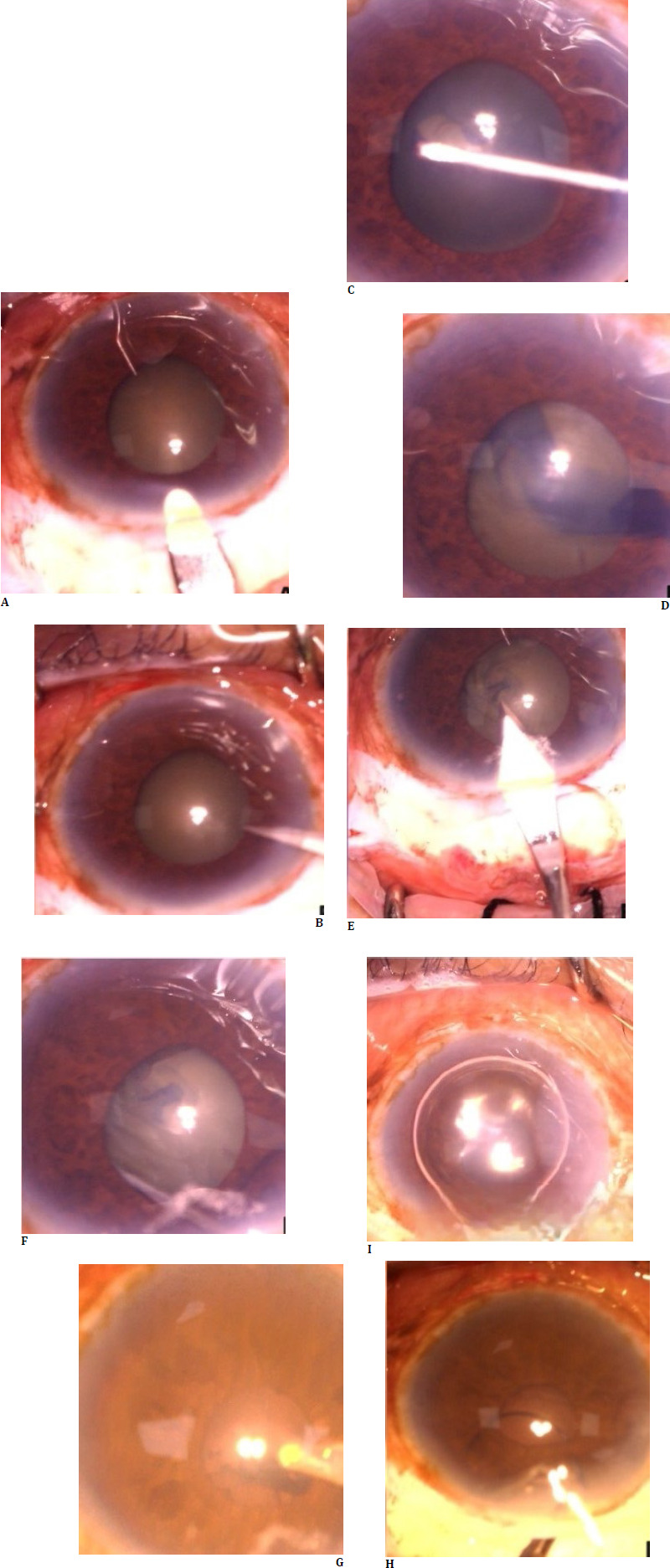
Steps of small incision cataract surgery in case 1, with steps represented sequentially. Capsulorhexis can be observed under the iris.

A 54-year-old patient with grade III nuclear sclerosis cataract with maximum pupil dilation of 4 mm underwent small incision cataract surgery. Routine surgery steps were performed meticulously, and a few tricks were used to manage the small pupil. Under the peribulbar block, a fornix-based conjunctival peritomy was performed, followed by a frown scleral incision and a constructed sclerocorneal tunnel. Continuous curvilinear capsulorhexis was performed after staining the anterior lens capsule with trypan blue dye. Special attention was made to achieve optimum 5.5 mm capsulorhexis by conducting it under the iris margin by guiding the capsular flap as per the tearing axis, even though the pupil was only 4 mm. Hydrodissection was performed by gently going along the anterior lens surface and under the iris as anterior lens margin was not made out. The nucleus pole was lifted into the anterior chamber and brought entirely into the anterior chamber using the visco cannula. Then, it was delivered using the visco-expression technique. Cortex aspiration was carried out using a Simcoe irrigation/aspiration cannula, and a rigid single-piece intraocular lens was implanted into the capsular bag (**Fig. 1**). Post-operatively, the patient had best-corrected vision of 6/6 with no complications noted until one year of follow-up.

## Case 2

**Fig. 2:**
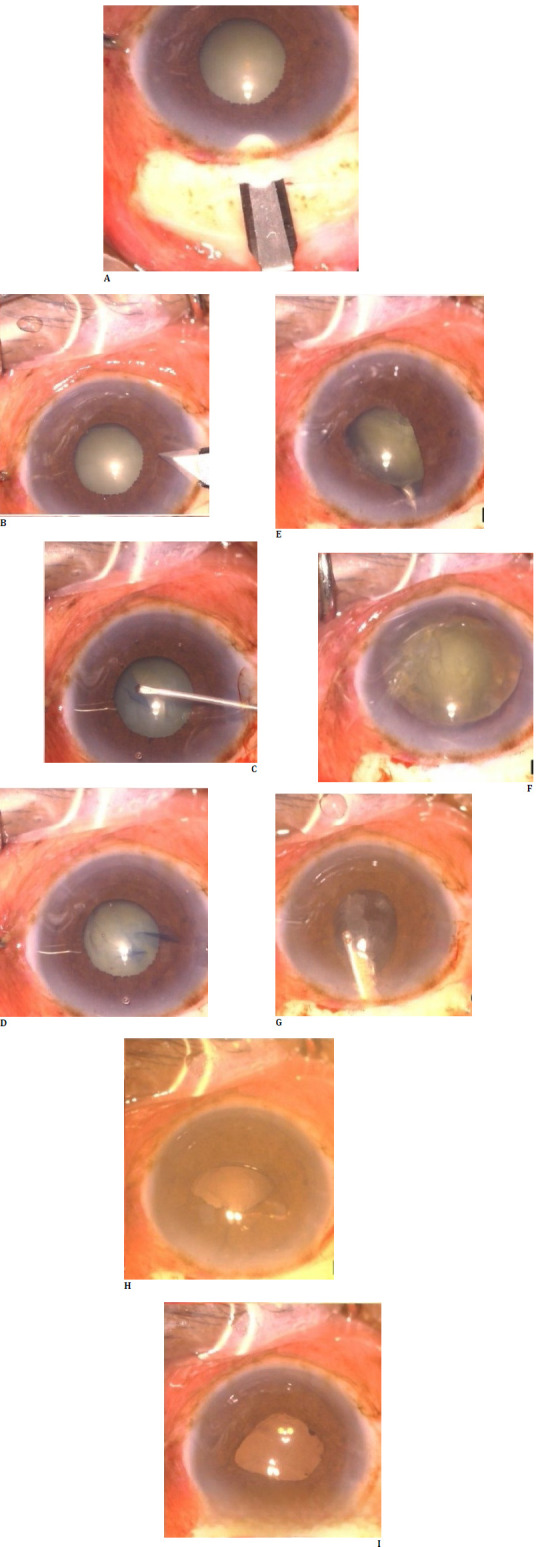
Steps of small incision cataract surgery in case 2, with steps represented sequentially. It can be observed that the pupil is round and regular, and the intraocular lens is well-centered at the end of the procedure.

A 48-year-old patient with maximum pupil dilation of 3.5 mm and grade IV nuclear sclerosis underwent uneventful small incision cataract surgery without using any mechanical devices. The patient had ocular trauma sustained a few decades back, and the other eye had pupillary dilation of seven mm. No phacodonesis or iridodonesis was noted, and the patient underwent steps as described in the first case with good post-operative recovery (**Fig. 2**).

## Case 3

**Fig. 3:**
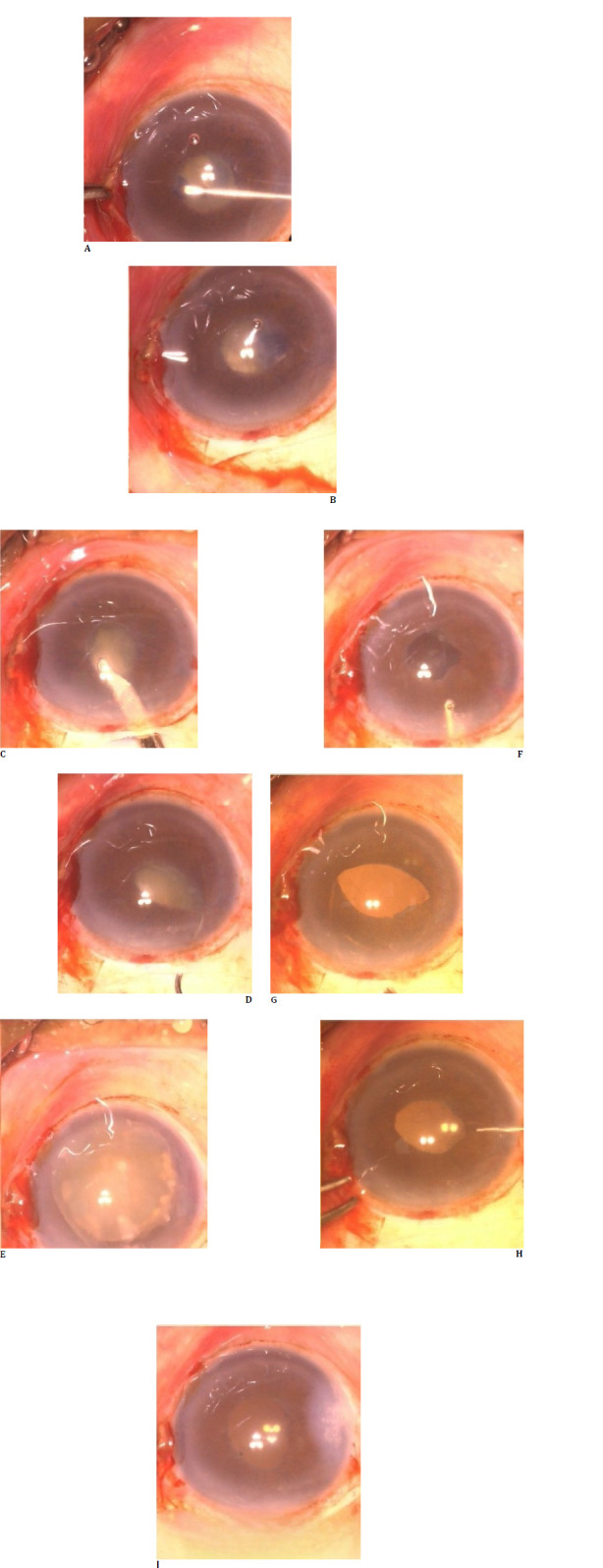
Steps of small incision cataract surgery in case 3, with steps represented sequentially. Anterior cortical decompression with Simcoe irrigation and aspiration cannula can be observed.

An 82-year-old patient who presented with visual acuity of hand movement and accurate projection of light had a mature cataract with a maximum pupil dilation of 3 mm. Anterior chamber depth was 2.12 mm, and intraocular lens power by optical biometry was 26 diopters. Pseudoexfoliation material is made out of pupillary ruff with dense arcus senilis. Considering the patient’s age and ocular surface status, specular microscopy found an endothelial count of 2100 mm2. The patient underwent small incision cataract surgery, and visco-adaptive agents were used judiciously with visco-dispersive agents to protect the corneal endothelium. After hydrodissection, loosened anterior cortical material was removed using Simcoe irrigation and aspiration cannula to decompress the lens content. The patient had good visual recovery and best-corrected vision 6/6 and underwent other eye cataract surgery (**Fig. 3**).

## Case 4

**Fig. 4:**
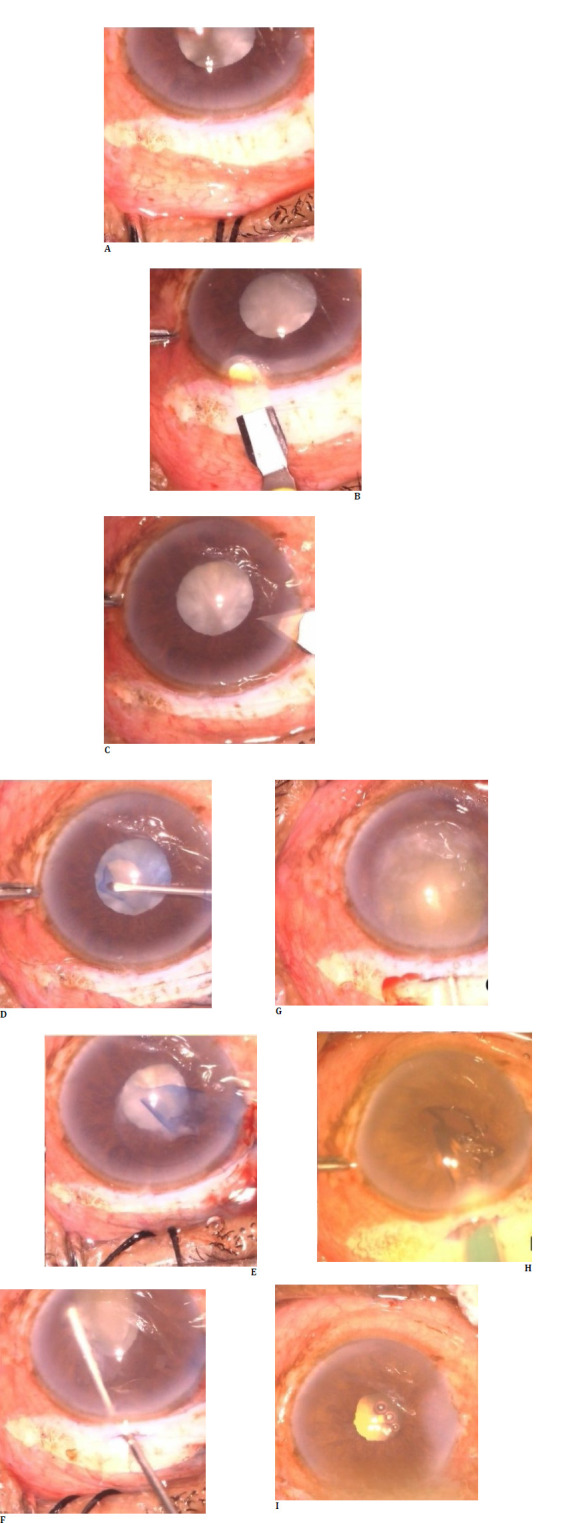
Steps of small incision cataract surgery in case 4, with steps represented sequentially. Cortex aspiration after nucleus delivery can be observed under the iris.

A patient in the sixth decade with visual acuity of hand movements and accurate light projection had a mature cataract with mild intumescence and a maximum pupil dilation of 3.5 mm. The patient’s other eye had cataract morphology suggestive more of a posterior polar cataract with onion ring configuration. The patient was taken up for small incision cataract surgery, with steps carried out to avoid any undue anterior chamber shallowing or capsulorhexis running away, considering the posterior lens capsule was weak. After performing capsulorhexis, swollen intumescent cortex materials were aspirated with a Simcoe cannula with any hydrodissection, and the nucleus mobilized into the anterior chamber using the flow of the Simcoe cannula. The posterior lens capsule appeared intact after nucleus delivery, so careful cortex aspiration was carried out, followed by lens implantation into the capsular bag (**Fig. 4**).

## Case 5

**Fig. 5:**
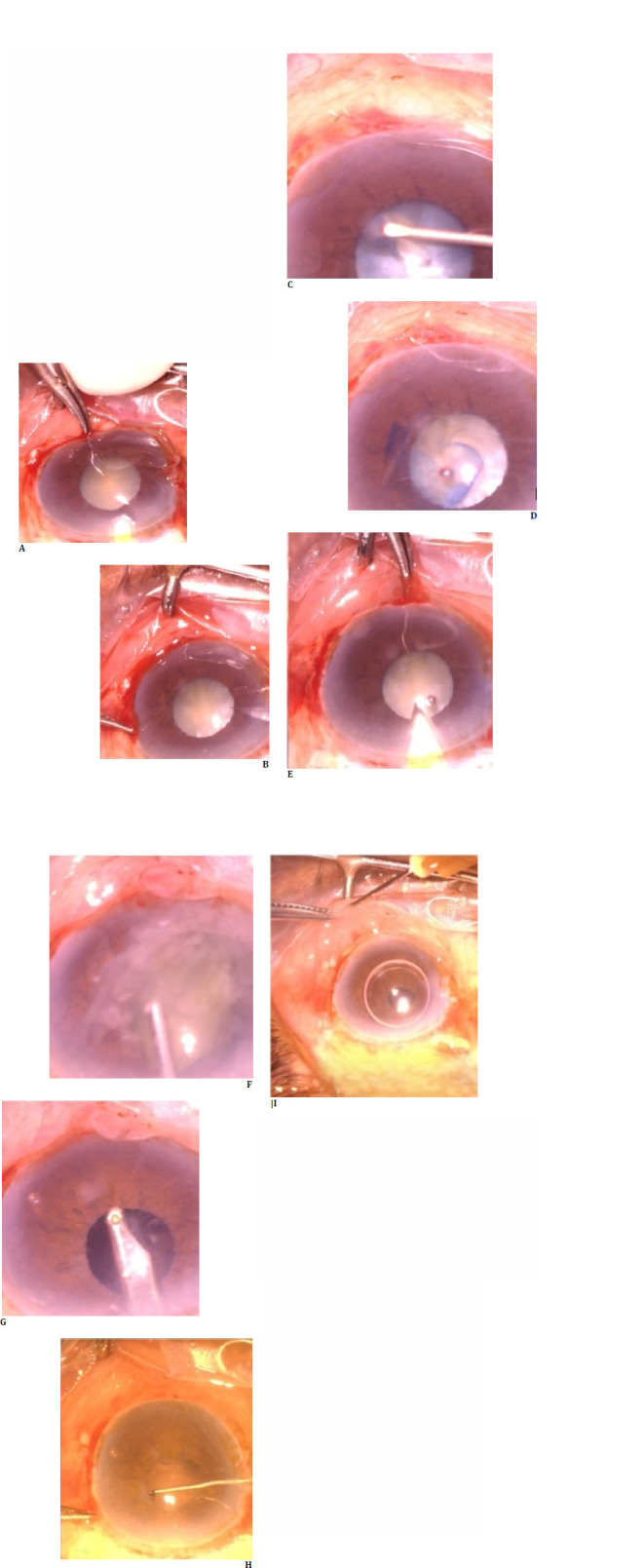
Steps of small incision cataract surgery represented sequentially in case 5

The patient was referred from a nearby eye hospital to our center with a history of hypotonus globe after giving a peribulbar block. A 50-year-old patient had visual acuity of perception of light and accurate light projection; intraocular pressure was 8 mm Hg. Axial length was 25 mm with mature intumescence cataract, and pupil dilation noted was 4 mm. B-Scan ultrasound revealed the presence of vitreous hemorrhage, so the patient was planned for small incision cataract surgery under sub-tenon’s anesthesia. The patient had an uneventful intra-operative course, and the intumescent cortex was managed using a Simcoe cannula before performing hydrodissection (**Fig. 5**). Post-operatively, the patient had clear cornea, and fundus examination revealed inferior vitreous hemorrhage and subsequent follow-up visits vitreous hemorrhage resolved. A laser barrage was done at the site of initial scleral perforation.

## Case 6

**Fig. 6:**
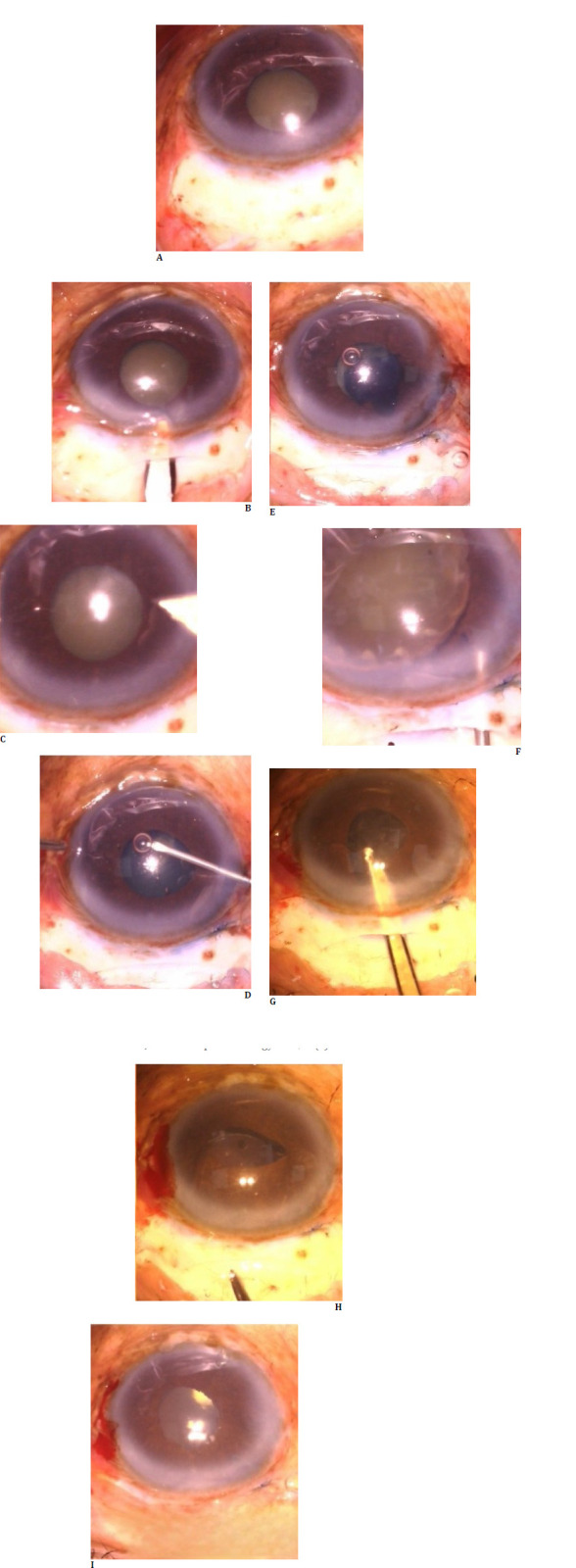
Steps of small incision cataract surgery represented sequentially in case 6

A 71-year-old patient with a maximum pupil dilation of 3.5 mm, pseudoexfoliation, and grade III nuclear sclerosis cataract underwent uneventful small-incision cataract surgery with the steps mentioned above and had a good visual recovery. No mechanical devices were used throughout the procedure, and the pupil remained round with its configuration maintained (**Fig. 6**).

## Discussion

Cataract is the leading cause of blindness worldwide, and cataract surgery is one of the most commonly performed procedures. Many advances are aimed at providing good visual recovery in patients, and still, small pupil can lead to poor visual recovery as it has a higher risk of complications [3]. Most cataract patients will have good pupillary dilation intra-operatively, and conditions like pseudoexfoliation, long-term glaucoma medications like pilocarpine, uveitis, previous ocular surgery, previous ocular trauma, diabetes mellitus, and intra-operative floppy iris syndrome are few causes for intra-operative poor pupillary dilation. Pseudoexfoliation is the major contributor to intra-operative small pupil, and the same has been found in a study by Drolsum et al. [4]. Various strategies have been used to improve pupillary dilation, and pre-operative use of non-steroidal anti-inflammatory agents is a routine among most cataract surgeries. Topical tropicamide, phenylephrine before surgery, and other cycloplegics like homatropine or cyclopentolate are commonly employed. Various intracameral mydriatic agents with local anesthetic combinations are available for commercial use, like Epi-Shugarcaine (Pioneered by Shugar in 2006, a combination of buffered lidocaine and phenylephrine) [5], a combination of intracameral tropicamide, phenylephrine and lidocaine (mydrane) is approved in some European countries. Mechanical devices include various pupil expanders like the Malyugin ring, B-Hex ring, I Ring, and Asian pupil expanders, which are commercially available. Various other techniques employed are stretch pupilloplasty, sphincterotomies, and iris hooks. Mechanical pupil expanders can be used in phacoemulsification and will not be beneficial in small incision cataract surgery. Fine et al. described the role of sphincterotomies in cataract surgery [6], and the procedure has its complications, significantly increased post-operative uveitis, glare, and rarely diplopia [7]. The authors report six cases with small pupil who underwent small incision cataract surgery without using mechanical pupil expansion methods. Technical expertise is needed for performing the few below-mentioned steps to achieve the optimum outcome:

- It is ideal to stain the anterior lens capsule with trypan blue dye and, if possible, to go under the iris and inject.
- Capsulorhexis is to be done under the iris either by lifting the iris with a sinskey hook or by following the tearing pattern of the capsule. Ideal-sized capsulorhexis is always aimed (5 to 5.5 mm).
- Gentle hydrodissection to be carried out by scratching the anterior part of the lens and going under the iris. A wave of fluid can be observed in mild-grade and denser cataracts. Forward prolapse of the nucleus can also be observed. A Simcoe cannula can decompress the capsular bag if the swollen or intumescent cortex is noted.
- Prolapse of nucleus needs support with viscoelastic agents and sinskey hook.
- Cortex removal is carried out using a Simcoe irrigation/aspiration cannula. A Sinskey hook can be used to access the cortex, and a careful watch for the anterior lens capsule is necessary.
- Placing the rigid intraocular lens into the capsular bag is also tricky, as one haptic has a high chance of settling in the sulcus. After placing the intraocular lens, the iris should be retracted using a sinskey hook or viscoelastic tip to confirm the location of an intraocular lens in the capsular bag.

## Conclusion

Cataract surgery is the most commonly employed procedure worldwide, and a small pupil is the primary reason for complications during the procedure. Small incision cataract surgery is performed widely in developing countries, and the authors report a case series of small pupil cataract surgery without using mechanical devices and tips for handling the case for optimum outcome.
